# Neurofilament Light Chain as Biomarker in Encephalitis

**DOI:** 10.3390/jcm13185416

**Published:** 2024-09-12

**Authors:** Sven Wellmann, Tobias Geis, Jens Kuhle, Verena Lehnerer

**Affiliations:** 1 University Children’s Hospital Regensburg (KUNO), Hospital St. Hedwig of the Order of St. John, University of Regensburg, 93049 Regensburg, Germany; tobias.geis@ukr.de (T.G.); verena.lehnerer@barmherzige-regensburg.de (V.L.); 2 Department of Neurology, University Hospital, University of Basel, 4001 Basel, Switzerland; jens.kuhle@usb.ch

**Keywords:** encephalitis, neurofilament, children, brain, neuron, NfL

## Abstract

Inflammation of the brain is called encephalitis and may result in acute and chronic brain damage. Encephalitis can be caused by various pathogens, especially neurotropic viruses, or can occur in the context of autoimmune diseases. Encephalitis is often difficult to diagnose and to monitor precisely during the course of the disease. Thanks to highly specific detection technology, components of the neuron skeleton, such as neurofilaments, can now be reliably quantified in the peripheral blood besides cerebrospinal fluid (CSF). Among them, neurofilament light chain (NfL) has demonstrated wide utility due to high preanalytical stability, robust diagnostic technology, and excellent reproducibility. We provide an overview of how NfL has advanced diagnostics in encephalitis and outline future avenues in research needs and possible clinical applicability of NfL in adults and children.

## 1. Introduction

Encephalitis is inflammation of the brain. The spectrum of symptoms and disease severity can vary largely. During onset, encephalitis may cause many different symptoms, which occur alone or in combination, including headache, fever, fatigue or weakness, aches in muscles or joints, confusion, personality changes, seizures or troubles with movements. Typically, over a period of hours to days, more severe symptoms occur, such as stiff neck, agitation, hallucination, seizures, changes in sight or hearing, loss of consciousness, and coma. Due to this variety of symptoms, encephalitis is difficult to diagnose clinically, and diagnostic biomarkers are needed to support clinical decision making.

## 2. Etiology of Encephalitis

In over 70% of cases, encephalitis is caused by bacterial or viral infections, which can be spread by insects, such as mosquitos and ticks [1]. In less than 30% of cases, encephalitis has non-infectious causes. In this case, the immune system attacks the patient’s own brain cells, which is known as autoimmune encephalitis [2]. For the causal treatment of encephalitis, rapid pathogen diagnosis is crucial. If the microbiological examinations are negative or an autoimmune disease is suspected, further autoimmune diagnostics will follow. In contrast to infectious encephalitis, in autoimmune encephalitis, symptoms may develop more slowly over a couple of weeks and are often unspecific, such as sleep problems, memory loss, muscle weakness, change in personality, or irregular movements. Typically, in autoimmune encephalitis, symptoms can be different for everyone, making clinical diagnosis, especially in autoimmune encephalitis, more difficult. In addition to the etiology of encephalitis, the question of the extent of brain damage is important for assessing acute and long-term impairment of those affected. In addition to clinical neurological diagnostics, neurophysiological methods, such as electroencephalography (EEG) and brain imaging using magnetic resonance imaging (MRI), diagnostics using suitable biomarkers in the peripheral blood offer an easily accessible, relatively inexpensive and serially feasible supplement.

## 3. Neurofilament Light Chain

**Figure 1:**
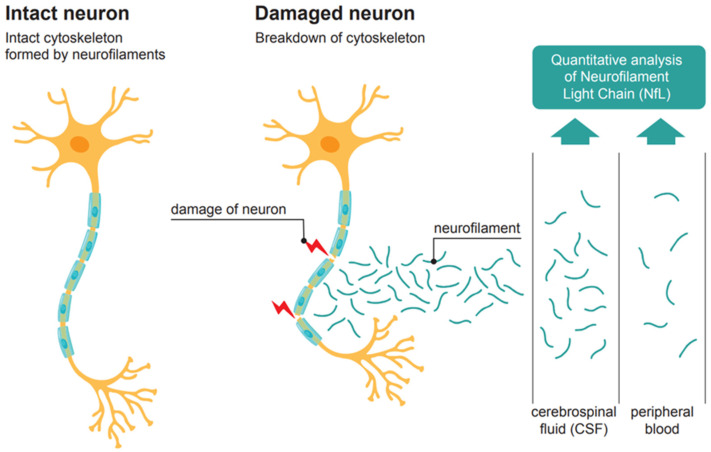
Neurofilament from neuronal scaffold to biomarker in cerebrospinal fluid and peripheral blood.

Neurofilaments are highly specific structural proteins of all neurons. They form the supporting skeleton of their axons. Different subunits of neurofilaments have been distinguished [3]. In axonal lesions, after pathological processes or trauma, neurofilaments leak into the extracellular space, from where they diffuse into the cerebrospinal fluid (CSF) and reach the peripheral blood, resulting in elevated concentrations (Figure 1). Among the four different neurofilament chains, the neurofilament light chain (NfL) appears to be particularly suitable for diagnostic purposes [3]. In peripheral blood, NfL has a half-life of about three to four weeks, and in both serum and plasma, NfL has been proven to be very stable with unchanged concentrations over a period of seven days at room temperature [4]. Moreover, in dried blood samples on filter cards, NfL can be quantitatively detected after significantly longer periods of time [5], and in frozen samples, NfL concentration remains stable for at least ten years [6].

Thanks to highly sensitive measurement using so-called single-molecular array technology (SIMOA) and automation of the measurement procedure, NfL can be precisely quantified in very low concentrations in plasma and serum (lower detection limit 0.8 pg/mL) out of a volume of just 25 μL. Via highly automated diagnostic setups, the result can be available within 45 min [7].

## 4. Physiological Factors Influencing Blood Neurofilament Light Chain

**Figure 2:**
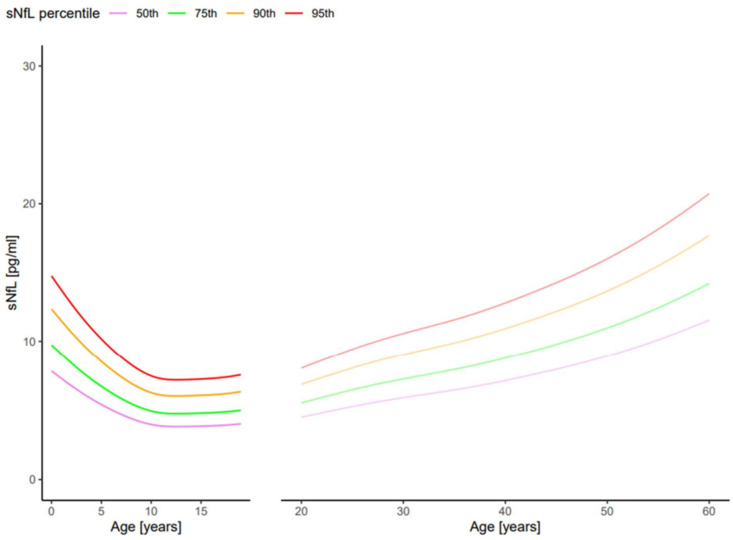
Serum neurofilament light chain (sNfL) percentiles derived from a pediatric cohort aged 0 to 20 years and from an adult cohort aged 20 to 60 years [8,12].

Pharmacodynamics and pharmacokinetics of NfL in CSF and peripheral blood are influenced not only by neuronal degradation (input) but also by the age of the patient, their body mass index (BMI) and their kidney function (output) [8]. While gender has no influence, an increased blood volume and an increased BMI lead to lower values (dilution effect) [9] and reduced kidney function to higher values [10,11]. In healthy neonates, NfL concentrations in serum (sNfL) comprise about 10 pg/mL. In children aged ten to eleven years, sNfL concentrations are the lowest in the entire lifespan at about 3 pg/mL. During aging, sNfl concentrations increase linearly to 10 pg/mL by about 50 years of age and thereafter rise faster, resembling a u-shaped curve during the whole life span as measured via SIMOA (Figure 2) [8,12]. To facilitate the diagnostic classification of individual NfL measurements in serum or plasma depending on age and BMI, Z-scores or percentiles (which are interchangeable) have been developed based on several 1000 healthy control persons. These can be used via a free internet tool from the University of Basel and allow for the quantification of the deviation of individual serum NfL concentrations from healthy controls (Z score 0 or 50th percentile) [8,12]. The web link to the free internet tool for adults is https://shiny.dkfbasel.ch/baselnflreference (accessed on 30 June 2024), and for children, https://shiny.dkfbasel.ch/baselnflreference-for-kids (accessed on 30 June 2024).

## 5. Neurofilament Light Chain in Infectious Encephalitis

In patients with infectious encephalitis, for example caused by the human immunodeficiency virus (HIV), herpes simplex virus (HSV), or herpes zoster virus (HZV), a direct correlation was found between NfL concentrations in the CSF and peripheral blood and the severity of the disease as well as the long-term neurological impairment [13,14,15]. Likewise, a severe acute respiratory syndrome coronavirus type 2 (SARS-CoV-2) infection can lead to encephalitis with consecutively increased sNfL concentrations [16]. Interestingly, it has also been shown in SARS-CoV-2 infections that increased NfL values occur without encephalitis—presumably due to dysregulation of the innate and acquired immune response with impairment of neuronal integrity [17]. This may explain why increased sNfL levels were also observed in those affected by mild to moderate COVID-19 disease [18,19]. Overall, it can be stated that COVID-19 is currently the best-studied viral disease in terms of NfL measurements, with over 100 specialist articles published in PubMed (as of the end of June 2024).

## 6. Neurofilament Light Chain in Non-Infectious Encephalitis

Non-infectious encephalitis typically manifests itself clinically through neuropsychiatric symptoms and is usually triggered by antibodies that target surface proteins, receptors or intracellular proteins of neurons [2]. Autoimmune-mediated encephalitis can arise in the context of a tumor disease or be triggered, for example, by drugs such as immune checkpoint inhibitors and subsequently lead to an increase in NfL [20,21]. If the autoantibodies are directed against surface antigens of the neurons, for example N-methyl-D-aspartate receptor (NMDAR), gamma-aminobutyric acid B receptor (GABABR), leucine-rich glioma inactivated 1 (LGI1), or contactin-associated protein 2 (CASPR2), the neurons usually remain intact. If autoantibodies are directed against intracellular antigens, for example Hu proteins, anti-collapsin response mediator protein 5 (CV2/CRMP5), protein Ma2, or glutamic acid decarboxylase 65-kilodalton isoform (GAD65), a T-cell-mediated immune response can be observed with the destruction of the cells and a significantly higher risk of epilepsy [22]. The greater the neuroaxonal damage is in such an autoimmune inflammation, the higher the increase in NfL levels in CSF and subsequently in peripheral blood, as was recently shown by the examples of anti-NMDAR encephalitis [23,24], GAD65 antibody-mediated encephalitis [25], neuromyelitis optica spectrum disorder (NMOSD) [26], and in myelin oligodendrocyte glycoprotein (MOG)-IgG-associated encephalitis of children [27]. NfL positively correlated with intensive care unit length of stay and outcomes scores [27] but in acute attacks, sNfL increase may occur with a delay after symptom onset [28].

Thus, the determination of NfL in the CSF or peripheral blood in non-infectious encephalitis can be helpful in terms of monitoring the severity of neuroaxonal damage and in monitoring the recovery phase. However, a one-time determination of NfL, for example in the initial diagnosis, does not seem to be an independent predictor of outcome in general, serial NfL measurements provide important predictive information.

## 7. Neurofilament Light Chain in Multiple Sclerosis

NfL is best studied as a neuroaxonal injury biomarker in the CSF and peripheral blood (serum and plasma) in pediatric and adult patients with multiple sclerosis, also known as disseminated encephalitis. This is an autoimmune disease in which the immune system attacks its own nerve tissue in the brain and spinal cord. The resulting multiple foci of inflammation can harden (sclerosis) over time. As of the end of June 2024, PubMed contained well over 500 research articles on NfL in multiple sclerosis, impressively demonstrating the diagnostic and prognostic relevance of NfL in easily accessible peripheral blood [29]. This biomarker is increasingly used as an adjunct and sensitive tool to capture disease activity in everyday clinical practice, and availability will increase, with several assays being developed using routine platforms already available in many institutions. Pediatric onset of multiple sclerosis (POMS) is a rare disease but an important differential diagnosis in many neuropediatric disorders [12,30].

## 8. Neurofilament Light Chain in Neurodegenerative Diseases Such as Alzheimer’s Disease

In general, neurodegenerative diseases have various causes, including inflammatory and non-inflammatory changes in the brain, with an increase known in those affected by autoimmune diseases [31]. All neurodegenerative diseases have the progressive loss of nerve connections (synapses) and the subsequent loss of neurons in common. Due to its relative frequency and the lack of or limited therapy options, Alzheimer’s disease has received large attention in recent years—also in connection with NfL. The longitudinal study of NfL in large cohorts has shown that an increase in serum and plasma NfL concentrations occurs several years before the onset of dementia symptoms [32]. Of note, single increased NfL measurements were less predictive than the actual change over time, area under the curve (AUC) or NfL trajectories [33]. In the early detection of Alzheimer’s disease, amyloid-based biomarkers in the CSF may be superior to NfL, but in serum and plasma-based diagnostics, serial NfL measurement is particularly advantageous in the early detection of dementia [34].

## 9. Conclusions and Outlook

In summary, neuronal cell damage can be specifically quantified by determining NfL in CSF, serum or plasma, regardless of the cause of encephalitis. Thanks to NfL’s high preanalytical stability, NfL determination is suitable for screening and therapy control in outpatient and remote settings. Latest analytical advances based on SIMOA technology with short turnaround time and the availability of NfL serum reference values from birth until advanced age put NfL at the forefront in the diagnosis and monitoring of encephalitis and other diseases affecting the nervous system. Especially in the field of pediatric patients, where the establishment of a diagnosis is more cumbersome, NfL holds great promise of advancing medical care. Whereas single determinations of NfL are helpful in screening for diseases or may help to rule out neuro-axonal damage, especially in the context of age and BMI-adjusted reference values, serial NfL assessments promise to be an effective way of monitoring treatments prospectively and over time. The ultrasensitive SIMOA technology was the first assay applied to establish serum NfL as a circulating biomarker in patients. More recently introduced assays such as Ella, a microfluidic cartridge-based immunoassay platform [35], will help to further advance the detection of NfL and other biomarkers and will help to reduce the costs of analysis and, as a result, increase availability.

## Data Availability

No new data were created or analyzed in this study. Data sharing is not applicable to this article.
